# In Pursuit of Enduring Inactivation: A Perspective on the Development and Study of Irreversible-Covalent Inhibitors

**DOI:** 10.1021/acs.biochem.6c00624

**Published:** 2026-10-05

**Authors:** Matthias Gehringer, David E. Heppner

**Affiliations:** † Department of Medicinal Chemistry, Institute for Biomedical Engineering, Faculty of Medicine, Eberhard Karls Universität Tübingen, Tübingen 72076, Germany; ‡ Department of Pharmaceutical and Medicinal Chemistry, Institute of Pharmaceutical Sciences, Eberhard Karls Universität Tübingen, Tübingen 72076, Germany; § Cluster of Excellence iFIT (EXC 2180) “Image-Guided and Functionally Instructed Tumor Therapies” Eberhard Karls Universität Tübingen, Tübingen 72076, Germany; ∥ Department of Chemistry, College of Arts and Sciences, The State University of New York at Buffalo, Buffalo, New York 14260, United States; ⊥ Department of Structural Biology, Jacobs School of Medicine and Biomedical Sciences, Buffalo, New York 14203, United States; # Department of Pharmaceutical Sciences, University at Buffalo, The State University of New York, Buffalo, New York 14214, United States; ∇ Department of Pharmacology and Therapeutics , Roswell Park Comprehensive Cancer Center, Buffalo, New York 14203, United States

## Abstract

Molecules that form irreversible covalent bonds with their targets are a mainstay of drug discovery and chemical biology research. Their ability to permanently inactivate an enzyme target provides a mechanism with significant potential to modulate disease phenotypes. This perspective discusses the trajectory of the field of irreversible enzyme inhibitors with an outlook on emerging methods, warhead structures, and sentiments presently directing practices in the discovery and design of the next wave of covalent agents.

## Graphical abstract

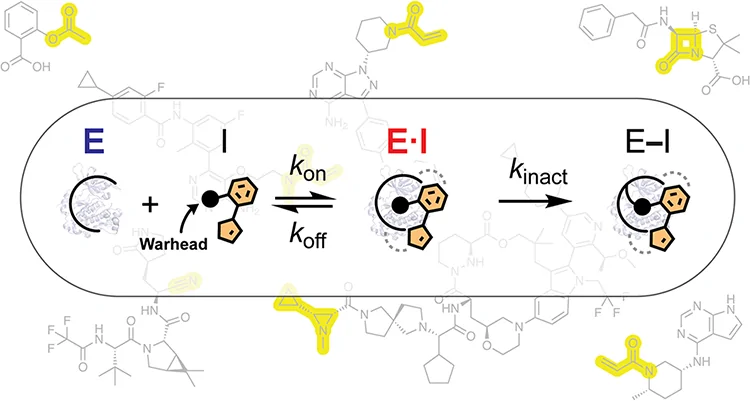

## Introduction

Gaining an edge on a pharmacological target can tilt the scales toward developing a drug to meet an unmet medical need. Since various confounding issues render highly sought-after targets especially challenging, this has motivated medicinal chemists to pursue innovations that push on long-standing “druggability” boundaries.^1,2^ Molecules that produce covalent bonds with their targets offer a productive twist on a conventional approach, which was initially met with resistance but has now become commonplace across academic and industrial research.^3,4^ It is now appreciated that ∼7% of all FDA-approved small-molecule drugs act through a covalent mechanism, which is likely to grow in the coming decades.^5^ In anticipation of the persistence and expanding attention to covalent inhibitors, we present a perspective on the modern status of the field and challenges that complicate their development.

## Evolution of the Field

The earliest notion of molecules that can irreversibly inhibit enzyme targets can be traced to work published in the late 1950s by Baker.^6^ In an expansive series of papers, Baker promotes the idea that nonclassical antimetabolites, which resemble an enzyme substrate but with a key structural change (e.g., methotrexate compared to folic acid),^7^ could serve applications where the compound would irreversibly bind to a structural feature adjacent to the active site. These so-called “Active-site-directed irreversible inhibitors” could be rationally designed and deployed as chemotherapies or protein structural tool compounds. Protein biochemistry was already well appreciated to allow for such inhibitor reactivity toward amino acid side chains, such as lysine amines, aspartic and glutamic acid carboxylates, histidine imidazoles, and, of course, cysteine thiols.^8–10^ In an early example, Baker puts forth a mechanism where endoalkylation of a substrate (S) ordinarily accepting the transfer of a reactive group (X) from a cofactor (F) in a ternary complex could be altered to a distinct group (B) to instead alkylate a nucleophilic component of the enzyme active site.^6^ The modern lexicon utilized today would describe X as a warhead comprising a certain electrophile capable of forming a strong and irreversible covalent bond.

While developing this innovative concept, irreversible covalent inhibitors were already being deployed as medicines, most notably Aspirin, which was on the market at the end of the 18th century and was proven to acetylate the noncatalytic Ser-529 on cyclooxygenase 1 in the early 1970s.^11^ Other examples of “Historical Covalent Drugs” that were medically efficacious and found to operate through covalent mechanisms include penicillin and other related β-lactam-containing antibiotics, which target a catalytic serine that is rendered highly nucleophilic through its incorporation into the catalytic triad of such hydrolase or transpeptidase enzymes. A paradigm shift from serendipitous to “Covalent by Design” occurred around the 2010s, ultimately resulting in the FDA approval of ibrutinib^12^ and afatinib^13^ for oncology indications. These approvals, and others that followed, helped to alleviate the persistent stigma concerning the apparent risks in pursuing covalent agents, which is now routinely done in small-molecule drug development campaigns.

We now appreciate that covalent agents are not restricted to enzyme inhibitors and can target various amino acids through diverse mechanisms. One major mechanistic quality involves the degree to which the covalent bond formed exhibits reversibility. Irreversible covalent inhibitors form a permanent adduct with the target residue, which contributes substantial binding energy and confers sustained pharmacological effects in cases where restoring the function of the target through expression of new protein is relatively slow. Reversible-covalent inhibitors, by contrast, engage the target through an equilibrium-governed covalent interaction. This enables fine-tuning of the inhibitors by modulating their unbinding kinetics and thereby strongly influencing their drug–target residence time. Notably, reversible-covalent protein–drug complexes have been shown to feature half-lives in the range between minutes to days,^14,15^ suggesting that the transition from reversible to irreversible covalency can be regarded as a continuum—a fact that is not sufficiently accounted for in the recent literature. One should note that the half-live of a reversible-covalent complex depends on various factors including the specific mechanism of bond formation and rupture, the stabilization of the covalent complex by the protein, and the availability of a suitable low-energy reaction path. While reversible covalency offers manifold opportunities for optimization, it also adds complexity. For example, although higher reactivity may be less problematic for reversible-covalent compounds due to the transient nature of the covalent adducts formed, their DMPK profiling may be non-trivial, perhaps due to compounds that can form “hidden pools” through reversible binding to bulk nucleophiles like glutathione (GSH) or abundant protein surface cysteines. It should also be noted that a covalent warhead may react in a reversible manner against one amino acid residue while reacting irreversible with another^8,9^ —a behavior that is sometimes erroneously interpreted as chemoselectivity for the irreversible partner, as this interaction is intrinsically easier to capture, e.g., by mass spectrometry-based methods.

The nucleophilic residue most commonly exploited in covalent inhibitor design is cysteine, whose thiol side chain exhibits p*K*_a_ values only slightly above physiological pH, giving rise to a highly nucleophilic thiolate species. The abundance of the thiolate reactive form can be substantially enhanced in specific protein microenvironments that increase the acidity of the residue side chain (i.e., lower the p*K*_a_ of the thiol).^16,17^ However, growing attention has been directed toward inhibitors that target other amino acid side chains, including lysine, tyrosine, histidine, glutamate, aspartate, as well as (catalytic and noncatalytic) serine and threonine, expanding the druggable covalent landscape beyond the subset of proteins that present a suitably positioned and reactive cysteine.

A key feature of covalent agents is an intrinsically reactive functional group meant to undergo a chemical reaction with the target amino acid to form the covalent bond. The example initially provided by Baker distinguishes this as the transfer group (B in Figure 1) but is referred to in contemporary literature as the “warhead” or “covalent reactive group” (CRG). Typically, the warhead is an electrophile that reacts with a nucleophilic amino acid side chain and is most desired to be both chemo- and site-selective. While chemoselectivity is derived from the chemical attributes of the warhead, site selectivity is achieved by tailored positioning of the warhead within the vicinity of the target residue through initial noncovalent binding to the target (vide infra). Here, well-tempered warhead reactivity ensures that the nonspecific covalent binding with other residues of the same type remains limited, typically relying on the relatively slow reaction of the warhead and such residue if a rate acceleration through noncovalent pre-binding is absent. Rate acceleration on target, a key feature of specific covalent inhibitors, is a highly intertwined process. It involves ligand affinity to promote the formation of the pre-reactive complex, preorientation of the reactive residues, and potential interactions with the target protein that lower the transition state for the formation of the potency-enhancing covalent bond. In this way, as proposed by Baker,^6^ covalent inhibitor reactivity on the target is similar to the enzyme-catalyzed process and not necessarily correlated with the intrinsic reactivity of the warhead (vide infra).

**Figure 1:**
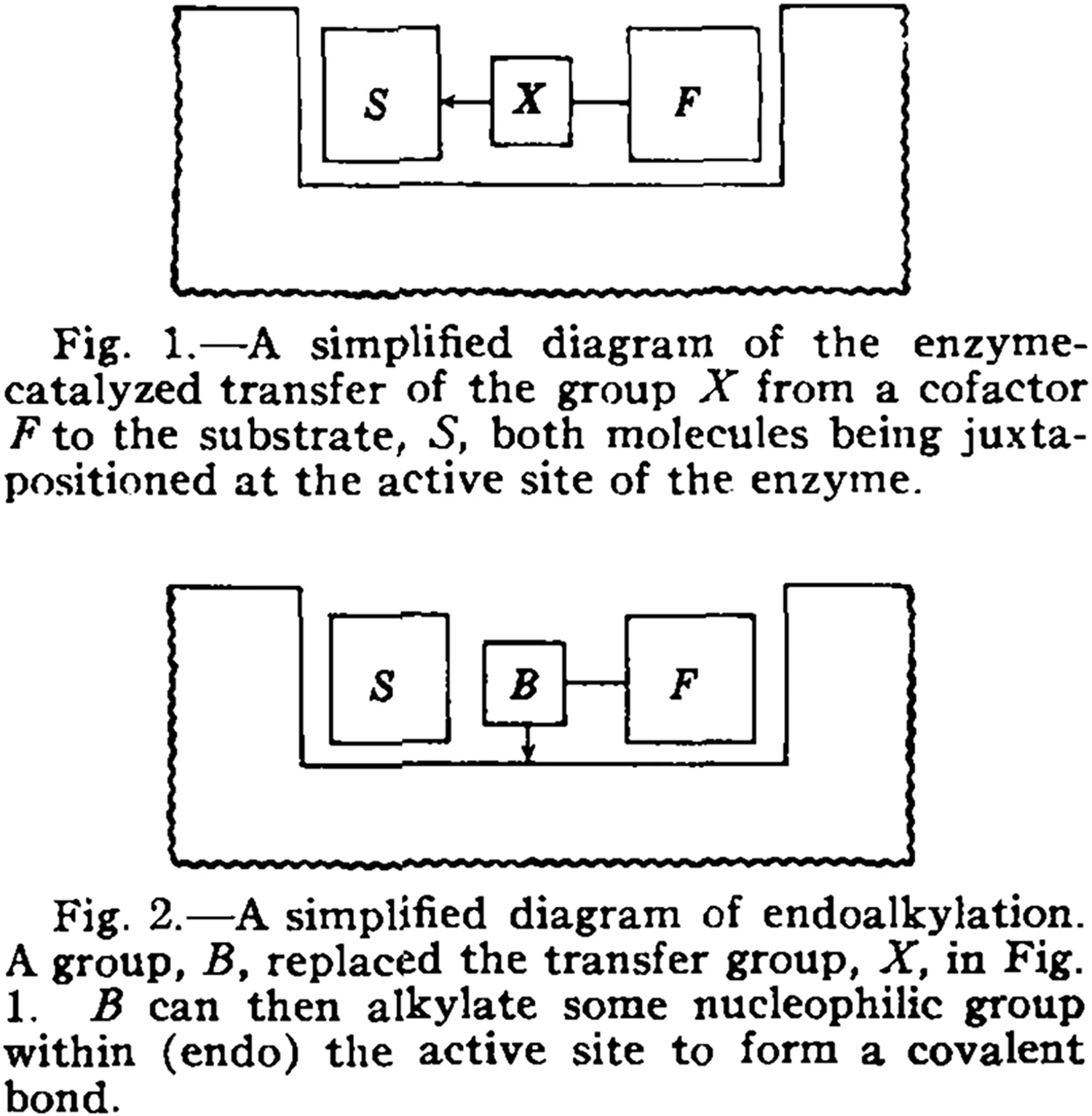
Schematic of the principle of an active-site-directed enzyme inhibitor put forward by Baker in ref.^6^ Reproduced with Permissions (License Number 6327720603763) Copyright © 1964 Wiley-Liss, Inc., A Wiley Company. Published by Elsevier Inc. All rights reserved.

To date, most covalent drug discovery efforts focus on cysteine, and by far, the most common warhead type encountered in this context is the α,β-unsaturated amide like acrylamide derivatives (note, for example, the chemical structures of afatinib, ibrutinib, and sotorasib in Figure 2). While these electrophiles offer significant advantages, including drug-like and tunable reactivity, favorable in vivo properties, and simple synthetic methods enabling their introduction to drug-like molecules at a late synthesis stage, it is not hard to appreciate that this has led to an overreliance on acrylamide-type warheads. There are intrinsic limitations to being overdependent on these warheads including limitations in the target scope of covalent agents since certain protein pockets—especially the more buried ones—may not be able to accommodate the steric properties and polar features of such moieties. Moreover, residues with distinct topologies [e.g., nucleophilic aromatic substitution (S_N_Ar)-based warheads or sulfur­(VI) bicyclobutane (BCB) strain-release warheads] may enable a more specific or productive approach to the target amino acid and circumvent metabolic liabilities such as double bond oxidation or amide cleavage associated with acrylamides.^18^ Given these well-appreciated complications, new warhead chemotypes targeting cysteines are highly sought after.^8,9,19–22^ Recent examples of non-canonical warheads with favorable profiles include the aforementioned S_N_Ar^23–25^ and strain-release warheads,^26,27^ heterocyclic Michael acceptors^28–30^ and also reversible-covalent chemistries like “double-activated” Michael acceptors (e.g., cyanoacrylamides),^14,31^ nitriles with different degrees of activation trough neighboring substituents^32^ or dihaloacetamides, which form hydrolyzable S_N_2-displacement products.^33,34^

**Figure 2:**
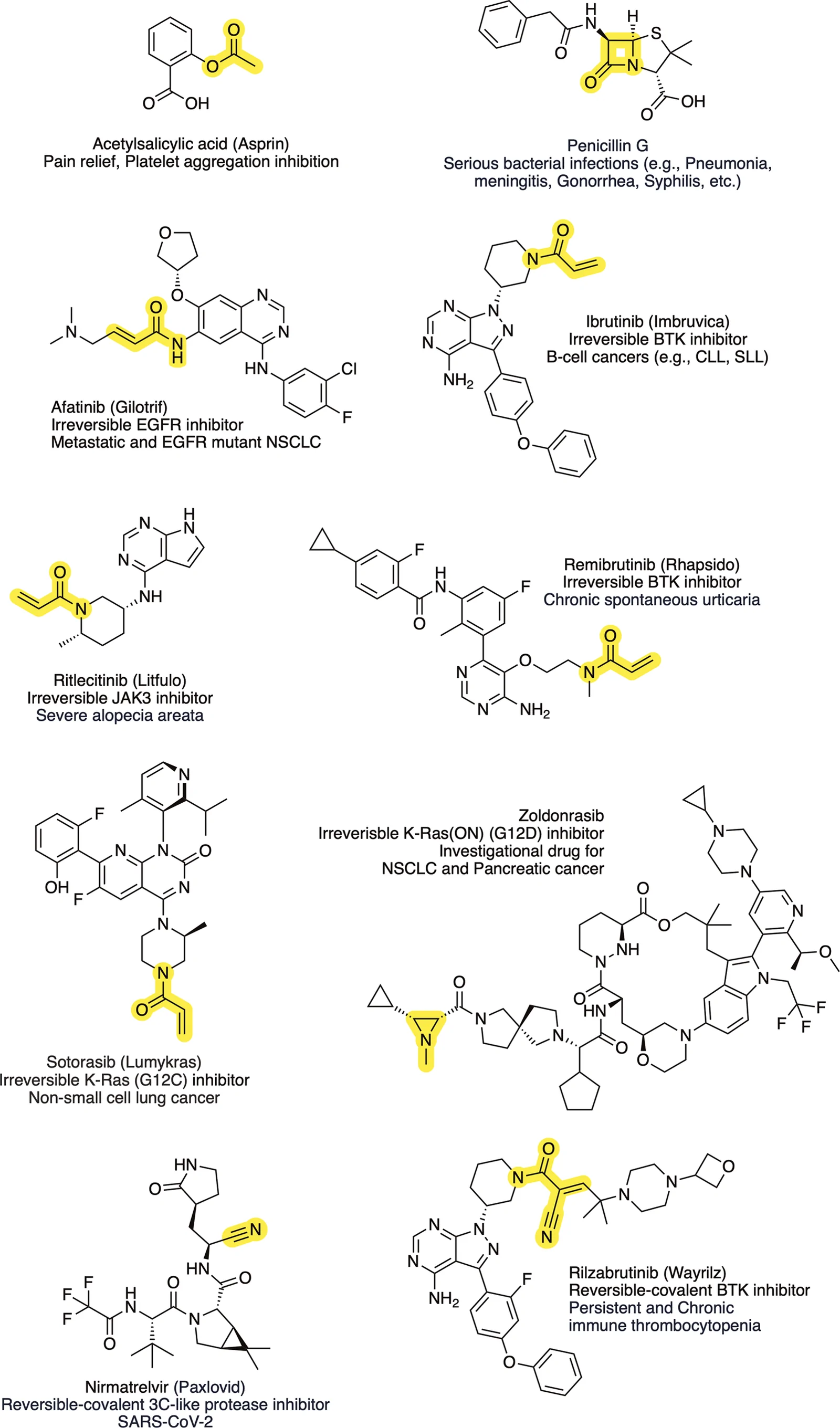
Representative covalent inhibitors and their chemical structures. The covalent warhead is highlighted in yellow.

An important emerging area is targeting amino acids beyond cysteine,^10^ since the latter has a comparably low occurrence in the proteome (∼2.3%) and tends to be, at least in the case of noncatalytic cysteine moieties, prone to mutation, which represents a common resistance mechanism against covalent drugs.^35–37^ Currently, a considerable shift in focus is being directed towards lysine with notable progress.^38,39^ Importantly, the lysine side chain (unperturbed p*K*_a_ ∼10.5) tends to be protonated at physiological pH making its ligandability very dependent on the acidification of this residue through unique elements within the secondary coordination sphere. Here, electrophiles such as sulfur­(VI) fluorides,^40^ acylation agents,^41,42^ squarates,^43,44^ and certain sulfone-based Michael acceptors^45^ have shown promise; however, the translational suitability of such moieties remains limitedly explored.

Reversible-covalent warheads targeting lysine residues typically form imine-type products that are stabilized through intramolecular interactions, as exemplified by the salicylaldehyde in voxelotor, which was originally approved as a sickle cell disease drug but is presently withdrawn.^39,46^ More unique warheads that operate through two-step mechanisms are emerging where initial reversible-covalent binding to a high reactivity electrophile such as an aldehyde is used to overcome the low abundance of nucleophilic (i.e., nonprotonated) form of lysine, followed by a second intramolecular reaction with a weaker electrophile that results in irreversible capture.^47–49^ Such mechanisms are used, e.g., in *o*-ethinylbenzaldehydes (EBAs)^47^ and analogous cyanobenzaldehydes (CNBAs),^49^ where the initial reversible attack can also occur with other (bivalent) nucleophiles while the irreversible cyclization is reserved for amines, which are distinguished by their trivalent nature. Emerging examples of N-acylated sulfonamide warheads and substituted sulfoximine Michael acceptors are also available and have been shown to exhibit specific bond formation for lysine over cysteine,^50^ including a tailored cyclization mechanism.^51^

Other amino acids, such as histidine, tyrosine, (noncatalytic) serine, or aspartate/glutamate, are also being increasingly explored.^8,9^ Although these efforts are still in their infancy, they led medicinal chemists to employ non-conventional chemistries, as exemplified by β-lactones,^52,53^ carbodiimide,^54^ diazoacetamide,^55^ and aziridines (Zoldonrasib in Figure 2)^56^ used to target KRAS­(G12D). While the latter two warheads are associated with DNA alkylation (see nitrogen mustard derivatives in the case of aziridines), Zoldonrasib from Revolution Medicines illustrates that aziridine reactivity can be tempered through steric shielding, resulting in a clinically viable covalent inhibitor that is currently undergoing evaluation in multiple clinical trials (e.g., NCT07621718, NCT06162221). However, while much remains to be discovered, significant challenges will have to be overcome to translate new covalent chemistries into therapeutic applications. Beyond issues related to synthetic accessibility, late-stage installation, aqueous and storage stability, additional hurdles include limited chemoselectivity, as well as largely unexplored DMPK and toxicological properties. Nevertheless, the examples discussed above reveal exciting new insights and directions that strongly motivate the expansion of warheads against amino acids that are presently recalcitrant toward reactivity with covalent agents.

### Discovery

Owing to their unique mechanisms, several techniques are available to enable the discovery of covalent inhibitors.^57,58^ One that is straightforwardly apparent is rational design by starting with well-defined reversible-binding compounds where three-dimensional structural information can be used to place covalent warheads to target nearby amino acid side chains. While such information has traditionally been mostly limited to target classes that can be characterized by X-ray crystallography, the rise of cryo-electron microscopy (cryo-EM) as well as in silico AI-based structure prediction,^59^ especially co-folding methods,^60^ have significantly broadened the scope of this approach. Alternatively, libraries of reactive compounds can be screened, which is referred to as “covalent first.” It is worth noting that many references also refer to this approach as “electrophile-first,” which we disfavor since the terminology is most commonly associated with screens of small covalent fragments where the electrophile is dominant, whereas covalent screening can also be performed using more complex molecules with substantial noncovalent binding contributions (vide infra). Such screenings are typically based on mass spectrometry methods detecting the irreversible formation of covalent adducts either on an intact protein or tryptic peptide level.^57^ Screening libraries originally comprised smaller collections of warhead-containing fragments with weak reversible-binding capabilities. In contrast, modern libraries typically comprise larger arrays of molecules with the intention to contain more elaborate, “lead-like” chemical structures.^61^ Such libraries typically deliver lower hit rates but higher quality hits featuring significant structural diversity, balanced physicochemical profiles, and more drug-like reactivity. Additionally, lead-like structures typically feature a stronger noncovalent binding component as well as consisting of architectures featuring synthetically tractable exit vectors, which facilitates their optimization and subsequent drug development. Notably, several successful biotech companies have been built around covalent-first screening pipelines, especially in the context of chemoproteomic profiling techniques,^62,63^ which allow for the assessment of ligand selectivity on a global proteome level early on in a program.^64^ On the other hand, an example highlighting the power of fragment approaches is the discovery of KRAS­(G12C) inhibitors through fragment disulfide formation (“disulfide tethering”)^65^ that allowed for hit identification and subsequent substitution of the ligand’s thiol group, ultimately enabling the development of an effective covalent inhibitor.^66^ Other techniques have been established, including *in silico* covalent docking (e.g., CovDock^67^ or co-folding^60^) as well as artificial intelligence-enabled structure prediction and covalent ligand design,^68,69^ in addition to covalent capture DNA-encoded libraries (DELs)^70^ and X-ray crystallography (fragment-based drug discovery) screening.^71,72^

## Kinetic Framework

Irreversible inhibitors (I) typically operate through a two-step inhibitory mechanism (Figure 3).^73–75^ First, an enzyme (E) binds the inhibitor exclusively through noncovalent interactions (enzyme-inhibitor complex, E·I) governed by the rate constants *k*_on_ and *k*_off_, which define the kinetics of noncovalent ligand association and dissociation. Subsequently, the warhead reacts with the enzyme to install a permanent linkage (inactivated enzyme, E–I). Importantly, these considerations extend beyond enzyme inhibitors and are equally relevant to other classes of irreversible covalent modulators. Since the covalent bond is permanently formed, the irreversible reaction step, i.e., a first-order process defined by *k*_inact_, makes the actions of these inhibitors inherently time-dependent. As such, accurate determination of the potency of an irreversible inhibitor requires one to measure the rate of formation of [E–I] (Figure 3A,B). Given the two-step mechanism (first reversible and second irreversible), applying the steady-state approximation allows for a rate law to be derived showing that the second-order rate constant *k*_inact_/*K*_I_ governs how quickly the inactivated E–I forms as a function of [E] and [I]. (Figure 3C). This *k*_inact_/*K*_I_ value is referred to as the inactivation efficiency and contains all three rates (*k*_on_, *k*_off_, and *k*_inact_). The *K*_I_ value referred to as the inactivation constant (Figure 3D). *k*_inact_ describes the “intramolecular” chemical reaction of E·I to E–I, which describes the maximal observed rate of inactivation, i.e., that rate observed once the target is saturated with the non-covalently bound inhibitor. It should be noted that although *k*_inact_/*K*_I_ is the key metric for assessing covalent inhibitor efficiency, it is a composite parameter and should best be interpreted, if possible, in the context of underlying *k*_inact_ and *K*_I_ values. Compounds with similar *k*_inact_/*K*_I_ ratios may exhibit markedly different behavior. For example, a high-affinity inhibitor with a slow *k*_inact_ may achieve an apparently favorable *k*_inact_/*K*_I_ despite exhibiting inactivation half-lives that are too long to be pharmacologically relevant. Another important consideration is that not all irreversible inhibitors exhibit the necessary saturation behavior depending on their affinity and reactivity, which is most common for molecules examined in covalent-first discovery methods. In such cases, alternative kinetic models must be employed, and the extraction of individual kinetic parameters becomes more challenging. As this scenario is relatively rare for classical covalent enzyme inhibitors, it falls beyond the scope of this article. The interested readers are referred to the cited literature for a detailed discussion.^76,77^

**Figure 3:**
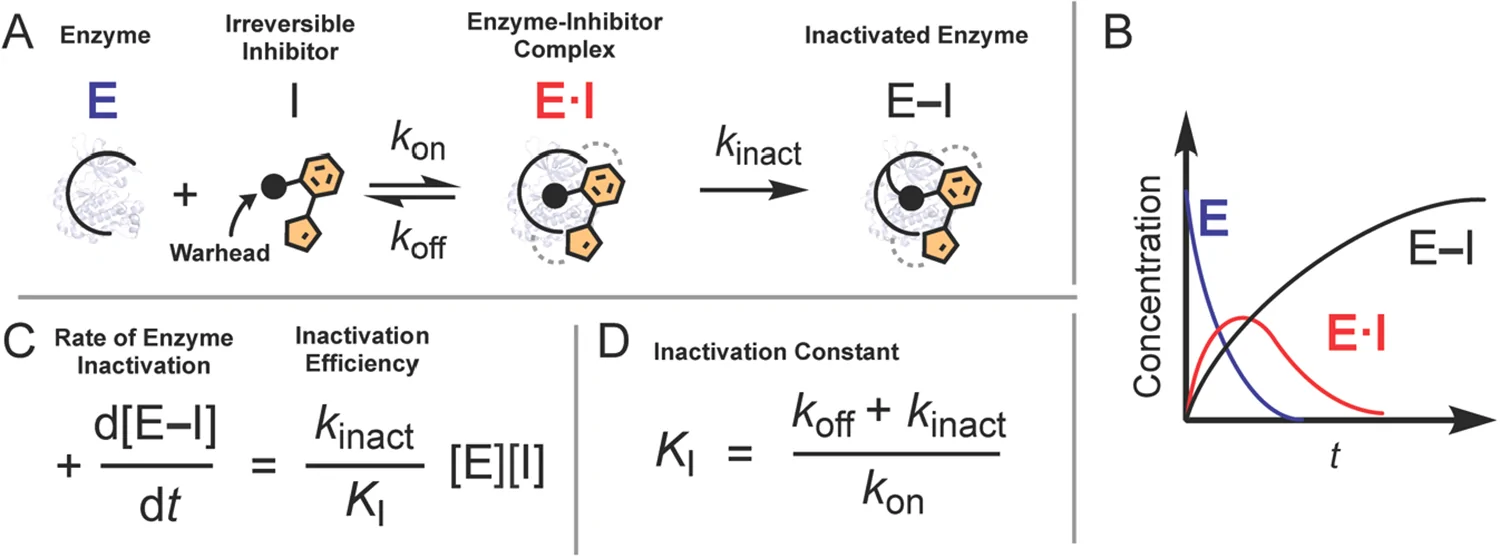
Kinetic framework of irreversible covalent inhibitors. (A) Two-step mechanism for irreversible covalent inhibitors. (B) Schematic of the time-dependence of the species involved in the inactivation of an enzyme by an irreversible inhibitor. (C) Definition of the inactivation efficiency (*k*_inact_/*K*_I_) is the second-order rate defining how quickly E–I is formed. Note: This expression as an illustration is mathematically simplified and only captures the kinetic behavior of E–I when [I] ≪ *K*_I_ (D) Definition of the inactivation constant (*K*_I_).

## Characterization Methods

One of the more complex landscapes in the covalent inhibitor field pertains to the methods used to determine their potencies. The time-dependent nature of the irreversible inhibition of an enzyme renders the determination of *k*_inact_/*K*_I_ rates the most reliable metric for assessing their relative activity toward developing structure-kinetic relationships (SKR). Standard equilibrium techniques that are used to determine *K*_i_, *K*_d_, and IC_50_ values fail to capture the irreversible nature of these molecules. It is worth acknowledging the existence of strong prevailing sentiments for the time-consuming and complex nature behind determining *k*_inact_/*K*_I_ values, especially in the context of the discovery of hit molecules.^77^ Successful drug discovery campaigns have utilized equilibrium characterization techniques in an attempt to overcome this challenge, with recent publications reporting “target engagement” TE_50_ values and “covalent ligand efficiency” as metrics that are inversely proportional to *k*_inact_/*K*_I_ and applicable in the context of covalent-first approaches.^77,78^ It is worthwhile to acknowledge the prevailing posture away from kinetic rate determination of many contemporary drug discovery scientists in the irreversible covalent inhibitor field. While exhaustive determination of *k*_inact_/*K*_I_ rates of all molecules in a development pipeline is not wholly necessary for delivering needed medicines to patients, there is no doubt in its superiority over other metrics in describing the potency of covalent agents.

Seeking *k*_inact_/*K*_I_ rates is practical in many cases, and this kinetic parameter can be obtained, for example, by quantifying inhibitor-dependent changes to the dynamics of substrate consumption or product formation.^76,79^ If monitoring a product, product curve analysis (PCA) can be obtained through the plotting of an inhibitory *k*_obs_ where the observation of rate saturation allows for the determination of individual *k*_inact_ and *K*_I_ values (Figure 4A).^80,81^ It is worthwhile to note that the expressions in Figure 4A are distinct from those noted in Figure 3C, as the former provides one route to experimentally determining *k*_inact_ and *K*_I_ values while the latter provides the mathematical framework for how *k*_inact_/*K*_I_ rates impact the dynamics of enzyme inactivation (i.e., how quickly the E–I is formed). Stopped-flow double-mixing, a well-established technique to determine rates, has been employed to determine individual *k*_inact_ rates in the characterization of covalent drugs targeting EGFR.^82^ While equilibrium techniques are not applicable, their time-dependence can be used to approximate *k*_inact_/*K*_I_ by comparing IC_50_ values measured at different incubation times (Figure 4B)^83,84^ although this technique has been attributed to quantitative inaccuracies that overestimate *k*_inact_ and underestimate *k*_inact_/*K*_I_.^76^

**Figure 4:**
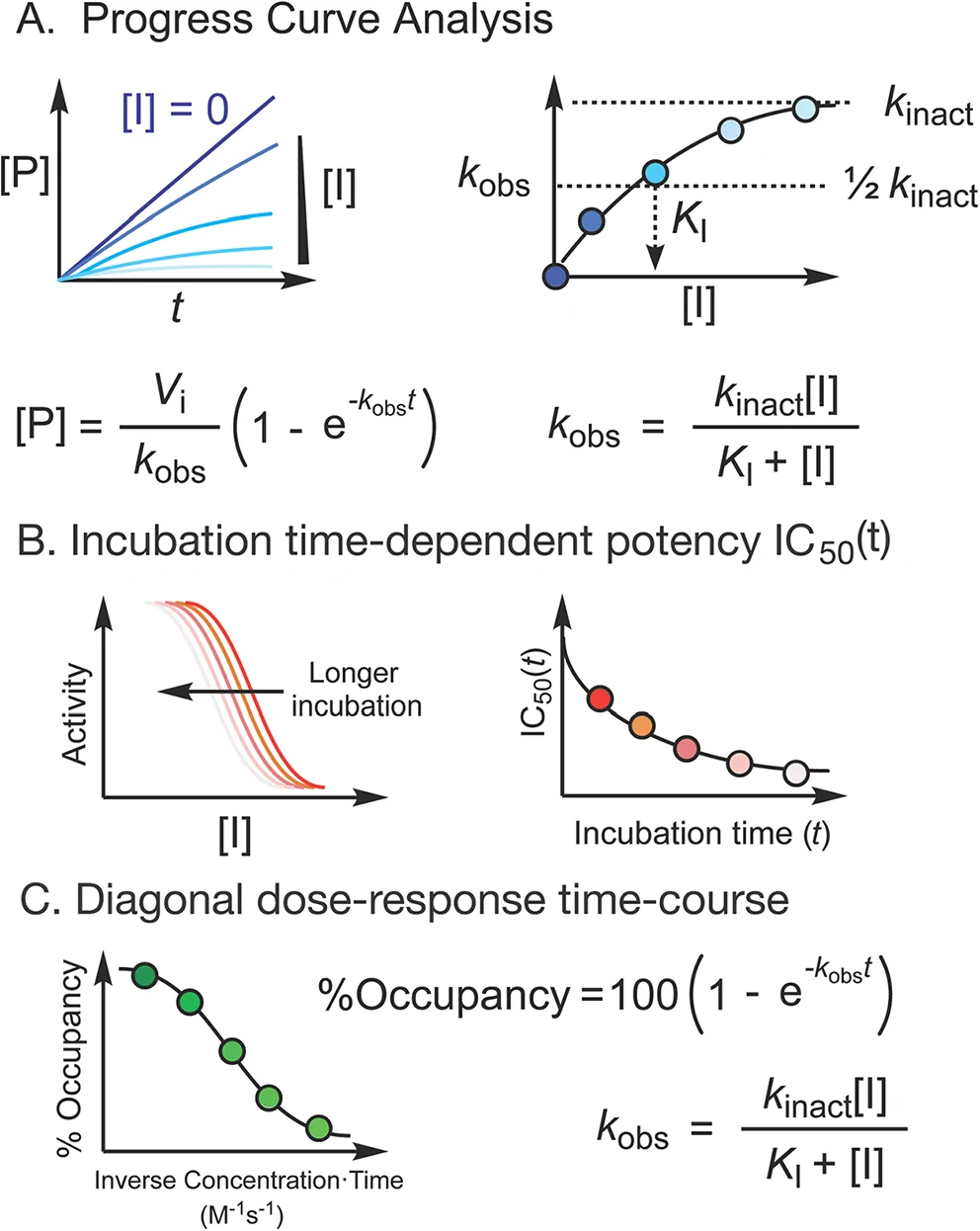
Representative illustrations of the diverse methods for *k*_inact_/*K*_I_ determination and associated mathematical expressions. (A) Progress curve analysis, (B) incubation time-dependent potency, and (C) diagonal dose-response time course.

Alongside the growing interest in covalent inhibitors, there is a desire to make potency assessments with increasingly higher throughput in drug discovery. Methods such as end point pre-incubation IC_50_ (EPIC-fit),^85^ and specialized protocols employing surface-plasmon resonance (SPR),^86^ and isothermal titration calorimetry (ITC)^87^ have recently emerged. Additionally, several investigators have extended time-dependent mass spectrometry (MS) techniques to monitor the formation of the E–I complex and quantify the dynamics through relative percent occupancy.^77,88,89^ One of the most comprehensive demonstrations of this was applied to characterizing KRAS­(G12C) inhibitors through solid-phase extraction-based RapidFire-MS amenable to robotic liquid handling capabilities, which drastically increases throughput.^88^ Additionally, a distinct biochemical method utilizing diagonal dose-response time course (dDRTC, Fig 4C), which involves determining intact MS percent occupancy in the context of a sampling of doses and incubation times, is poised to deliver *k*_inact_/*K*_I_ values with simplified analysis and reduced experimental effort, enabling broad applications in drug discovery.^89^ Alternatively, an extension of fixed-time-point target engagement (TE_50_) values, which can be related to percent occupancy, is proposed to be a sufficient alternative to *k*_inact_/*K*_I_ rate determination for non-saturable kinetics, as is common especially for early compounds identified through covalent-first discovery.^77^

Innovative techniques have also emerged, showcasing the ability to determine the actions and properties of covalent drugs in complex biological settings. For instance, fluorescent activity-based probes allowed for *k*_inact_/*K*_I_ rate determination of penicillin-binding proteins for assembling cellular SAR.^90^ A recently reported method utilizing binding kinetics on a proteome-wide scale is covalent occupancy kinetic enrichment via proteomics (COOKIE-PRO).^91^ This technique promises a probe-focused determination of *k*_inact_/*K*_I_ values for all cellular targets of covalent inhibitors, enabling the kinetic characterization of a covalent inhibitor with proteome-wide insights.^91^ Additionally, unprecedented whole-animal details of covalent inhibitor action were developed with the volumetric Clearing-Assisted Tissue click CHemistry (vCATCH) approach that enabled in vivo drug distribution mapping across the adult mouse.^92^

As is the case in a highly impactful and dynamic field, the emergence of a multitude of techniques offers diverse avenues of discovery but leaves questions regarding the comparability or consistency of their findings. While much in this regard is presently unknown, it is worth appreciating that these measurements are complex and not necessarily comparable from method to method.^80^ Recent analysis found that different reports of EGFR inhibitor *k*_inact_/*K*_I_ values with identical inhibitors and targets were often significantly different, especially when different measurements were employed. Additionally, several assay variables, such as how inhibitor solutions are prepared, can alter the observed *k*_inact_/*K*_I_ rates unpredictably, further complicating decision-making in drug discovery. It is highly likely that many of the methods mentioned earlier suffer from similar issues of comparability, and investigators should be cautious with respect to making claims based on data from different literature reports or in-house methods. The best approach is to maintain tightly consistent assay conditions and liquid preparation steps. Experimental reference compounds are absolutely necessary, as values from the literature should never be assumed to be sufficient for gauging internal consistencies when using *k*_inact_/*K*_I_ rates to inform SKR. All buffer additives (e.g., reductants, enzyme stabilizers, and detergents) should be tested for their potential impact on *k*_inact_/*K*_I_ rates of reference compounds. Our experience generally suggests that trends in *k*_inact_/*K*_I_ can be captured in cross-study comparisons; however, some caution should be applied, especially in the context of making design choices.^80^ Naturally, the same applies to other assay readouts. Given the expansive nature of the diverse techniques described above, it is worth pausing to consider their limitations. A general perspective is likely true that as a method incorporates more biological elements (e.g., cell-based and proteomics), accurate and reproducible determination of covalent rates will be intrinsically challenging.^90–92^ Future studies are necessary to best frame results from cutting-edge techniques in context with the guidance provided here and elsewhere in the literature.

## Kinetic Rates in the Optimization of Irreversible Covalent Inhibitors

As is common in drug discovery, a leading series gradually evolves from iterative changes to a starting chemical scaffold, often referred to as design-make-test-analyze cycles, toward a goal potency and other profile parameters. At the earliest stages, one is typically most interested in increasing potency through gradually strengthening the binding of an inhibitor for the target. For fully reversible-binding inhibitors, it is well appreciated that orders of magnitude changes in IC_50_ or *K*_i_ indicate significant improvements that are being made to potency. In fact, it is more appropriate to express IC_50_ values as log-transformed pIC_50_ to best capture trends in potency for reversible-binding molecules.^93,94^ These trends in potency guide medicinal chemists toward the construction of project-defining structure-activity relationships (SAR). However, irreversible covalent inhibitor potency is governed by a change to the *k*_inact_/*K*_I_ second-order rate constants, which is a distinct metric from the more common equilibrium or thermodynamic measurements.

Seeking to better appreciate how to interpret *k*_inact_/*K*_I_ in the context of a drug discovery campaign, a simple series of experiments was conducted utilizing the epidermal growth factor receptor (EGFR) as a model drug target.^95^ Here, the results of time-dependent PCA biochemical assays were compared to antiproliferative effects in cell line models in EGFR L858R/T790M versus WT systems.^96^ Correlations between these experiments showed that orders of magnitude faster *k*_inact_/*K*_I_ rates were consistent with significant improvements in cellular potency, as expected; however, as the inhibitors became increasingly more active, the ability to select the “best” inhibitors became increasingly challenging. An examination of selectivity for the L858R/T790M mutant versus WT EGFR allowed for proper rank-ordering of covalent inhibitors; however, the differences between the *k*_inact_/*K*_I_ rates were more modest (∼2-4-fold). This is consistent with several earlier studies showing that relatively small changes in *k*_inact_/*K*_I_ correlated with important differences in biological activities.^97,98^ Interestingly, similar modest-fold changes were found between on- and off-target kinases when characterizing ibrutinib in cells with COOKIE-PRO.^91^ These observations allowed for the formulation of guidance for how *k*_inact_/*K*_I_ rates can be interpreted both in early phases of drug discovery, where orders of magnitude rate improvements are likely to be achieved, as well as later lead optimization stages, where smaller-fold changes, in combination with tuning various other relevant properties, can be used to fine-tune inhibitor profile parameters. In this context, it should also be noted that target turnover is a critical determinant of covalent inhibitor action as discussed below.

## Complications in Drug Discovery

The trends and results discussed above are likely to be determined by several complicating factors. One interesting implication is how increasingly faster *k*_inact_/*K*_I_ values do not seem to lead to increasingly improving potencies in cell line models.^99^ One such explanation may be that inhibitors exhibiting increasingly faster *k*_inact_/*K*_I_ rates in biochemical assays do not actually exhibit faster kinetics in cells because of limitations of diffusion within the complex cellular milieu.^100^ This very well could be the case for the EGFR inhibitors examined in this work, as their *k*_inact_/*K*_I_ rates approach or exceed 10^6^ M^–1^ s^–1^. The diffusion limit in an intracellular environment can be assumed to be lower than that in aqueous solution. Additionally, recent work showcases that the first noncovalent and second covalent steps are inversely coupled at the diffusion limit, akin to *k*_cat_ and *K*_M_ for enzymes.^101^ This indicates that if a covalent drug is operating at the diffusion limit, the affinity for the target is offset by the on-target reactivity (i.e., *k*_inact_). In fact, it could be of great utility to strive for optimizing structures toward faster *k*_inact_ rates if this can be achieved without increasing intrinsic reactivity substantially. In this context, it is important to point out that the intrinsic reactivity of a covalent inhibitor (sometimes referred to as *k*_chem_) is not necessarily correlated with *k*_inact_, a misconception still frequently found in the literature. The overall sense is that covalent inhibitor optimization should be considered a balancing act of both affinity and *k*_inact_ kinetics rather than optimizing both in isolation (i.e., focusing exclusively on reversible binding).^101^

Another underexplored aspect in covalent drug discovery is the dynamic nature of the target. Reversible agents elicit their pharmacological effects by almost fully binding their targets in a biological environment, which is conventionally articulated in the context of occupancy theory or occupancy-driven pharmacology. Much the same can be said for irreversible covalent inhibitors, except in this case, the target binding is permanent and time-dependent. The added benefit from this scenario is clear, as the biological effects—once the compound irreversibly binds—are not tied to the pharmacokinetic behavior of the molecule. However, it is required that the covalent inhibitor can achieve full target binding within the context of the dynamics of target expression. In this manner, covalent inhibitor levels and their respective *k*_inact_/*K*_I_ rates must outcompete the apparent “resynthesis” of new active target by cellular transcription and translation, as otherwise most advantages of irreversible inhibition would be lost. This adds increased complexity to covalent inhibitor discovery as rapidly resynthesized targets will require fast inactivation,^102^ which needs to be balanced in the context of potential off-targets that may be resynthesized much slower. Indeed, differences in target turnover of different proteins may, to a certain degree, override differences in *k*_inact_/*K*_I_ rates and may thus significantly alter selectivity profiles when moving from biochemical to cell assays. On the positive side, disease targets with intrinsically slow recycling kinetics may be superiorly targeted with covalent agents, as is the case for gamcemetinib targeting MK2 in autoimmune disorders.^103^ Even with a relatively low *k*_inact_/*K*_I_ of ∼5 × 10^3^ M^–1^ s^–1^, the low turnover of MK2 in the range of 1–2 days, together with the compound’s suitable pharmacokinetic profile, enabled high target occupancy in vivo. Covalent target (and off-target) occupancy of irreversibly acting compounds can be assessed and monitored by chemoproteomic approaches, which typically rely on covalent competitor probes. Notably, it has been demonstrated that BTK inhibitors like acalabrutinib^104^ or remibrutinib^105^ are cleared rather fast but show sustained target occupancy due to the slow resynthesis of BTK. Such dynamics may also be at play in EGFR-driven cancer cells and are another potential explanation for the lack of improved cellular effects with increasingly faster covalent inhibitors.^106^ Given this added complexity, further studies and methods need to address the resynthesis of targets, also considering different cell types and cellular states.

## Structural Biology Considerations in Understanding Covalent Inhibitor Action

Structural interrogation of an enzyme-ligand complex is common in drug discovery and highly informative in characterizing covalent inhibitors.^107^ X-ray crystallography still remains the most commonly utilized technique, with increasing structures coming from cryo-electron microscopy (cryo-EM). Typically, a covalent inhibitor is structurally characterized with the protein side-chain covalent bond formed, which is useful for confirming the resulting geometry and positioning of the warhead after reaction. However, it could be seen as only a portion of the needed information to guide efficient optimization. It is easy to note that the reversibly bound complex (E·I) may be of greater use compared to the inactivated enzyme (E–I), where the positioning of the warhead and binding modes of the inhibitor are best understood. There is always the possibility that the enzyme protein structure alters its conformation upon covalent bond formation. For example, we recently obtained X-ray crystal structures of various covalent inhibitor-bound kinases with target cysteines located in loop regions (e.g., MKK6: PDB IDs 8P7J and 8PM3, MELK: PDB ID 9F31; ULK1: PDB ID 9F32).^64^ Retrospective comparison of these crystal structures indicated substantial rearrangements of the cysteine-containing loops relative to (noncovalent) PDB structures. It may be most useful for structural biologists to characterize non-reactive inhibitor analogues to best appreciate these factors. Molecular insights of the E·I complex are likely to contain vital perspectives into drug design, facilitating the extrapolation of the trajectory for the covalent bond formation step that defines *k*_inact_.

Covalent modification has also been shown to destabilize certain proteins in cells, particularly when it occurs within structurally disordered regions of the target. This phenomenon has led to the development of “covalent destabilizing degraders” for conventionally challenging proteins such as MYC,^108^ β-catenin,^109^ and AR-V7,^110^ an oncogenic androgen receptor splice variant lacking the regulatory ligand-binding domain. Covalent protein modification can also alter thermodynamic protein stability, as assessed by differential scanning fluorimetry (DSF) and related thermal shift assays. Although many covalent ligands substantially stabilize their target proteins, cases of destabilization, manifested as a reduction in protein melting temperature, have also been reported.^111–113^ A recent study suggests that, at least in the context of covalent fragments, such destabilization may correlate with high intrinsic reactivity and limited target specificity.^114^ Alternatively, thermodynamic destabilization has also been observed for more elaborate ligands exhibiting greater selectivity and/or modest intrinsic reactivity, in some cases accompanied by substantial structural perturbations of the target protein.^64,111,113^

It should be noted that structural interrogation of any protein-ligand complex comes with caveats that can potentially mislead drug discovery. All crystallographers are dutifully aware that the conditions to produce protein crystals lie far from physiological conditions and therefore produce artificial environments that risk complicating structural analysis. One such variable involves the overall packing of the protein into a lattice amenable to atomic-resolution X-ray diffraction.^107^ Results concerning the structural basis of AZD9291 (osimertinib) targeting the T790M gatekeeper-containing EGFR kinase showcase how the nature of the overall protein structure can influence the conformation of inhibitor binding, specifically dependent on whether the enzyme–ligand complex is made through crystal soaking or cocrystallization.^115^ This example strongly suggests that pre-forming the covalent complex may be worth the extra costs in cocrystallization protocols. Of course, one of the principal features of a structure is the observation of the covalent bond, which is not always evident in the structure.^116^ In some instances, covalent inhibitors do not show productive bond formation often due to unknown factors.^117^ For reversible-covalent inhibitors, the molecule can be found to coexist as both the covalent and noncovalent complexes.^31,118^ It is worthwhile also noting that observing the target covalent bond is not guaranteed due to a number of limitedly explored factors. For example, high-energy X-ray radiation may result in the radiolytic cleavage of the ligand-target covalent bond, or unexpected side reactions may deactivate the covalent warhead of the covalent inhibitor prior to forming the covalent bond with the target.^119–121^

These different confounding issues strongly motivate the search for improved computational methods for applications in covalent drug discovery. Specifically, this involves detailing the properties of covalent bond formation as well as time-dependent techniques to better understand the factors that may aid in the optimization of a drug candidate.^122^ We anticipate that computational methods must be developed alongside complementary experimental techniques (e.g., assays of protein mutations or structural alterations to the agent chemical structure) to afford robust outcomes that do not misdirect structure-guided drug design. This is especially the case for covalent inhibitors, where their complex mechanisms of action add additional intricacies and confounding variables.

## Summary of Key Concepts and Definitions

Several core concepts have been introduced in the course of this perspective, and various definitions are included here as a summary of key concepts. Because irreversible covalent inhibition proceeds through a two-step mechanism, i.e., reversible binding followed by covalent bond formation, its behavior cannot be captured by a single potency value in the way that equilibrium-governed reversible inhibition often is. Instead, a distinct vocabulary has developed to separate the contributions of initial binding affinity, intrinsic chemical reactivity, and the downstream biological consequences of sustained target engagement.^81^ The terms below are collected here for reference and are used consistently throughout this perspective.

- **Affinity**. The strength of the initial, noncovalent binding interactions between the inhibitor and the enzyme prior to the formation of any covalent bond (E + I ⇌ E·I).
- **Inactivation Constant** (*K*_**I**_). The concentration of inhibitor [I] needed to reach the half-maximal rate of inactivation (i.e., [I] at 1/2 *k*_inact_) (units = M).
- ***k*_inact_**. First-order rate constant for covalent bond formation defines the maximum rate of inactivation at infinite [I] (units = s^–1^).
- **Inactivation Efficiency** (*k*_**inact**_ **/** *K*_**I**_). The overall second-order rate constant that describes the rate of enzyme inactivation (overall E + I → E–I) into a single efficiency measure, allowing for potency and selectivity comparisons of irreversible enzyme inhibitors (units = M^–1^ s^–1^).
- **Intrinsic Reactivity** (*k*_**chem**_). The inherent chemical reactivity of an electrophilic warhead toward a nucleophile in solution is independent of molecular recognition within a target binding site. It is commonly assessed using small-molecule model nucleophiles, such as glutathione, to provide a target-independent measure of the warhead reactivity.
- **Target Turnover**. The rate at which the target enzyme is resynthesized in the cell that governs how quickly a covalently inactivated enzyme pool is replaced by newly synthesized, active enzyme.
- **Pharmacological Outcome**. Net biological or clinical effect of covalent target engagement.

## Conclusions and Outlook

The pursuit of covalent inhibitors has never been more intense and is likely to increase given the advent of new drugs and chemical biology methods. Their impact is unquestionable, yet much of what is applied in their development is not systematic or consistent across the groups. For instance, intrinsic reactivity toward the abundant thiol-containing molecule glutathione is a mainstay of this field for cysteine-targeting molecules,^74^ and recent studies have reaffirmed that additional considerations of warhead susceptibility of glutathione *S*-transferase enzymes provide improved principles for inhibitor optimization.^123^ Various aspects of covalent inhibitor properties have also been underexplored. In any case, the expanding array of clinically effective drugs speaks to their influence. Despite the expanding arsenal of warheads operating through diverse mechanisms targeting noncysteine side chains, the development of molecules that go beyond cysteine comes with significant additional challenges, including warhead selectivity, unknown ADMET properties, and stability issues. The reliance on covalent bond formation, especially in the context of reversible-covalent inhibitors, produces challenges in modeling the PK/PD behavior of a drug, which is essential for translational research. An expanded body of covalent probes and drugs is anticipated, and fundamental research into the actions of these agents is required to better position this field for lasting and more predictable impacts. The future of the covalent inhibitor field is likely to depend on its ability to accept common approaches in the development of new drugs to enable efficient and broad development of needed therapies.
